# Topical Tranexamic Acid Reduces Blood Loss in Minimally Invasive Total Knee Arthroplasty Receiving Rivaroxaban

**DOI:** 10.1155/2017/9105645

**Published:** 2017-12-19

**Authors:** Shih-Hsiang Yen, Po-Chun Lin, Bradley Chen, Chung-Chen Huang, Jun-Wen Wang

**Affiliations:** ^1^Department of Orthopaedic Surgery, Kaohsiung Chang Gung Memorial Hospital, Chang Gung University, College of Medicine, Taoyuan, Taiwan; ^2^Institute of Public Health, National Yang Ming University, Taipei, Taiwan; ^3^Kaohsiung Chang Gung Memorial Hospital, Chang Gung University, College of Medicine, Taoyuan, Taiwan

## Abstract

**Background:**

It is unclear whether topical (intra-articular) or intravenous TXA reduces blood loss in minimally invasive TKA patients receiving a direct oral anticoagulant for thromboprophylaxis. This study is to investigate whether TXA given intravenously or intra-articularly is effective in reducing blood loss in minimally invasive TKA patients using rivaroxaban for thromboprophylaxis.

**Methods:**

Ninety-three patients who underwent primary minimally invasive TKA were divided into placebo group (30 patients) that received saline both intravenously and intra-articularly, intravenous (IV) group (31 patients) that received 1 g TXA intravenously, and topical group (32 patients) that received 3 g TXA in 100 ml saline intra-articularly. All patients received oral rivaroxaban of 10 mg daily for 14 days postoperatively.

**Results:**

*p* < 0.001 and *p* = 0.041. The mean total blood loss was 1131 mL (567–1845) in placebo, which was higher than that in the IV group (921 mL; range, 465–1495; *p* = 0.014) and the topical group (795 mL; range, 336–1350; *p* < 0.001). The total blood loss did not differ between the IV and the topical group (*p* = 0.179).

**Conclusion:**

This prospective, randomized, controlled trial demonstrated an equal efficacy of TXA in blood conservation when administered intravenously or topically in minimally invasive TKA patients receiving rivaroxaban for thromboprophylaxis.

## 1. Introduction

Total knee arthroplasty (TKA) is associated with substantial blood loss in the range of 1000–1800 mL, which may necessitate allogenic blood transfusion [1–3]. Previous studies reported a transfusion rate ranging from 10 to 38% after standard TKA [1, 4, 5]. Transfusion carries significant risks of cardiopulmonary embarrassment, disease transmission, immunological reaction, and postoperative infection [1, 3, 6].

Tranexamic acid (TXA), an inhibitor of fibrinolysis, if intravenously administered, is reportedly effective in terms of decreasing blood loss after standard [7–9] as well as minimally invasive TKA [10, 11]. Although several meta-analyses have demonstrated the safety and efficacy of intravenous administration of TXA in primary TKA patients [12–16], the increasing thromboembolic risk from administration of a large dose of TXA (20 mg/kg) with or without continuous infusion remains a concern [6].

In view of the efficacy of topical application of TXA to the surgical field associated with dental and cardiovascular surgical procedures in terms of reducing bleeding, intra-articular application of TXA before wound closure at the time of TKA has been advocated recently, following promising results obtained by Wong et al. [17]. In theory, topical application of TXA during TKA will be safer in terms of thromboembolic risk than systemic administration. The efficacy of topical TXA for reducing blood loss in primary TKA has been confirmed in clinical trials [18–20] and a meta-analysis [21]. Recently, a comparison of topical and intravenous TXA administration in primary TKA performed by Gomez-Barrena et al. [19] demonstrated an equal efficacy in terms of reducing blood loss, with no safety concerns. However, all of the above-mentioned trials or studies of topical application of TXA were performed in standard TKA patients.

The minimally invasive technique for TKA is commonly used in orthopedic surgery, the advantages of which are a smaller wound, less pain [22, 23], faster rehabilitation [22, 24], quicker return to work [25], and possibly a less amount of blood loss [23]. Our previous clinical trial showed a blood conservation level of 15% upon intravenous administration of TXA in minimally invasive TKA patients [11]. However, the efficacy in terms of reducing blood loss by topical application of TXA in minimally invasive TKA patients is still unclear. Moreover, the patients in most of the studies that have analyzed the blood conservation effect of topical TXA administration in TKA were not receiving modern oral anticoagulants for thromboprophylaxis.

Specifically, we asked (1) Whether topical or intravenous administration of TXA is effective in reducing total blood loss after minimally invasive TKA patients receiving rivaroxaban for thromboprophylaxis and (2) whether there is difference of postoperative blood loss and transfusion rate between the two blood conservation regimens in minimally invasive TKA patients.

## 2. Materials and Methods

This study was registered in the public register at Clinical Trials.gov (NCT02453802) and supported by our institutional grant (CMRPG8D1051).

The sample size was calculated according to the method of Benoni et al. [26], who carried out a prospective randomized trial for calculation of perioperative blood loss. Assuming a mean difference in total blood loss of 225 mL or greater between the 2 groups, to obtain a statistical power of 0.90 and an alpha error of 0.05, 30 patients are required in each group. With the expectation that 10% of patients would be excluded from the analysis and 10% would be lost to follow-up, 100 patients were enrolled.

**Figure 1:**
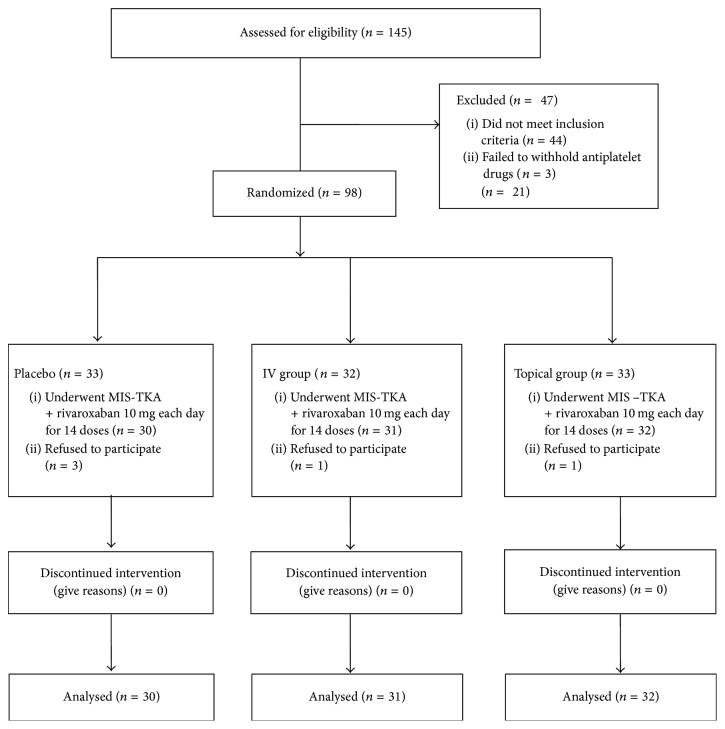
Flow chart of patient selection and analysis throughout the study. IV: intravenous, MIS: minimally invasive surgery, and TKA: total knee arthroplasty.

Between August 2015 and April 2016, a consecutive series of 145 patients aged 40 years or more who underwent unilateral primary minimally invasive TKA were assessed in terms of their eligibility for inclusion in this study. The exclusion criteria were patients with a documented history of thromboembolic disease, cardiovascular disease (myocardiac infarction or angina), stroke, coagulopathy, lifelong warfarin treatment for thromboembolic prophylaxis, impaired hepatic or renal function (impaired hepatic function was defined as liver enzyme level, AST or ALT, which is more than twice normal range, history of liver cirrhosis, elevated total bilirubin level, or coagulopathy (INR < 1.3); and impaired renal function was defined as GFR < 55 ml/min/1.73 m2, which is relative contraindicated for chemical venous thromboembolism and venography), and patients with an allergy history to tranexamic acid or concomitant use of protease inhibitors of human immunodeficiency virus, or fibrinolytic agent that contraindicated the use of rivaroxaban and preoperative anemia (a hemoglobin level of ≤10 g/dl). All patients were instructed to withhold aspirin, nonselective cyclooxygenase inhibitors, and antiplatelet agents for at least 7 days prior to surgery. We excluded 44 patients based on the exclusion criteria. Three patients did not withhold antiplatelet drugs for 7 days before surgery, and five patients declined to participate after group allocation. Thus, the remaining 93 patients (23 male and 70 female) were enrolled in this study (Figure 1). Patients were assigned randomly into 3 groups: a placebo group (*n* = 30), a topical TXA group (*n* = 32) and an IV TXA group (*n* = 31) by an independent research assistant who was not involved in the study via a computer-generated method. The research assistant placed the study medications into sequentially numbered opaque sealed envelopes, which were kept in our Clinical Trial Pharmacy. On the day of operation, the research assistant sent the sequentially numbered envelopes taking from the research pharmacy to the operating room according to sequence of the surgery. The envelope was opened and the study medications were prepared by an anesthetist not involved in this study. The study medications were all identical. The patients, surgeons, research assistant, and nurses in charge were all blind to the randomization until the complete data were all collected.

The dose of intravenous TXA used in this study was chosen based upon the results of our previous study, which indicated that a 1 g TXA intraoperative bolus injection is effective for blood conservation after TKA [11]. The dose of topical intra-articular TXA in this study was set according to the results of a study by Wong et al. [17] who demonstrated that a topical intra-articular dose of 3 g TXA in 100 mL of saline resulted in a high efficacy of blood conservation in TKA.

The preoperative characteristics of the patients, including age, body mass index (BMI), preoperative hemoglobin (Hb) level, hematocrit (Hct), prothrombin time (PT), activated partial thromboplastin time (APTT), platelet count, and American Society of Anesthesiologists (ASA) grade [27], were all similar in the 3 groups, the exception being the gender distribution (Table 1). All knee surgeries were performed or supervised by the same surgeon (JWW) using a minimidvastus approach as described by Haas et al. [24]. All the participants received general anesthesia. A pneumatic tourniquet was applied and inflated to a pressure of 350 mmHg before incision and deflated after skin closure. All TKAs were primary and unilateral and were cemented using the same prosthesis (Nex-Gen, Legacy Posterior Stabilized Prosthesis; Zimmer, Warsaw, IN, USA). An intramedullary guidance system was used for femoral cutting and an extramedullary guidance system was employed for tibial cutting. The femoral canal was routinely plugged with bone. Two intra-articular drainage tubes were placed and connected to a vacuum bag without compression of the bag for 12 hours, followed by full compression of the bag until removal. The volume of blood drained was recorded during the first 16 postoperative hours and from 24 to 36 hours according to the time of drain removal. Patients in the placebo group received saline twice, 20 mL intravenously 10 minutes before skin closure and 160 mL intra-articularly via the drain after capsule closure before deflation of the tourniquet. Patients in the topical TXA group received 10 mL of saline intravenously 10 minutes before skin closure and 3 g (60 mL) TXA (Transamin 50 mg/mL; China Chemical and Pharmaceutical Co, Taiwan) in 100 mL saline intra-articularly via the drain after capsule closure. Patients in the IV group received 1 g (20 mL) TXA intravenously 10 minutes before skin closure and 160 mL saline intra-articularly via the drain after capsule closure. The drainage tubes were clamped for 1 hour after injection of the study medications. The tourniquet was not deflated until skin closure and application of a compressive dressing. All patients received intravenous prophylactic antibiotic therapy consisting of 1 g Cefazolin preoperatively followed by 1 g every 8 hours for 3 doses postoperatively. Standard VTE prophylaxis was prescribed in all patients, by oral intake of rivaroxaban (Xarelto, Bayer Shering Pharma AG, Wuppertal, Germany) at 10 mg once daily starting on postoperative day (POD) 1 for 14 doses. All patients were required to get out of bed with support on POD1 to decrease the incidence of DVT [28]. No other modalities such as compressive devices of the leg or foot pumps were used. The discharge criteria were (1) clear operative wound without discharge, (2) the range of motion of the knee being 90 degree or more, (3) patients able to ambulate with walker support, and (4) no symptoms of anemia or venous thromboembolism.

The primary outcome measured was the estimated total blood loss. Hemoglobin (Hb) and hematocrit (Hct) levels were measured on PODs 1, 2, and 4. The blood volume was assumed to have normalized on the fourth postoperative day. Total blood loss was calculated according to the method of Nadler et al. [29] using the maximum postoperative decrease in Hb level adjusted for the weight and height of the patient. The formula can be summarized as

(1) Total  blood  loss=Total  blood  volume×maximum  reduction  in  Hb  levelmean  Hb  level+volume  transfused.

The secondary outcomes were the rate of perioperative blood transfusion, the rate of deep-vein thrombosis (DVT), wound complications, visual analogue scale (VAS) on POD 1, the length of hospital stay, and the range of motion of the knee. The trigger for allogenic transfusion of red blood cells was set at a Hb level of 8 g/dl in healthy patients, or between 8 and 9 g/dl in patients with clinical symptoms and signs of acute anemia. In patients with cardiovascular disease, the transfusion threshold was set at a Hb level of 9 g/dl. The amount and rate of blood transfusion and the length of hospital stay were recorded in all patients.

The patients were followed-up at the clinic postoperatively after 2, 6, and 12 weeks. All postoperative conditions, including wound erythema, hematoma, infection, bleedings, and blisters, were recorded. We inspected the appearance of the operated knee, and if the swelling was noted, the knee circumference was measured 1 cm proximal to the upper pole of the patella. If the circumference of operated knee increased ≧3 cm compared with the opposite knee, it was defined as intraarticular hematoma formation. All wound complications requiring return to surgery because of hematoma and infection within 30 days of procedure were recorded. The presence of DVT was detected upon measurement of thigh or leg edema of >3 cm as compared with the contralateral leg in association with calf tenderness or tightness [30]. Ascending venography of the leg was performed using the Rabinov and Paulin technique [31] if symptomatic DVT appeared. Computerized tomography of the chest was performed if there was a suspicion of pulmonary embolism. All radiographic images were interpreted by an independent radiologist.

## 3. Statistical Analysis

One-way ANOVA was used to determine differences between the three groups in the demographic characteristics, preoperative clinical data, total blood loss, postoperative drainage amount, postoperative hemoglobin level, length of hospital stay, VAS, range of motion of knee, and wound length. If the one-way ANOVA result was significant (*p* < 0.05), post hoc tests were performed to confirm where the differences occurred between the groups.

Differences in descriptive data, including gender, ASA level, incidence of DVT, blood transfusion rate, and safety outcome, between the three groups were compared using the chi-square test or Fisher's exact test. An intention-to-treat analysis was used in patients who undergone planned surgery, had taken study medications, and withdrew blood for preoperative and postoperative Hb levels. All statistical comparisons were made using the Statistical Package for Social Sciences (SPSS) (version 18; SPSS Inc., Chicago, IL, USA).

## 4. Results

The postoperative results are shown in Table 2. The wound length under full extension of the knee did not differ between the 3 groups. The Hb levels on POD1, POD2, and POD4 were significantly higher in the topical group than in the placebo group (*p* = 0.005, *p* < 0.001, and *p* < 0.001, resp.). There were no significant differences in Hb level between the placebo group and the IV group on POD1 and POD2, but the Hb level on POD4 was higher in the IV group than in the placebo group (*p* = 0.043). The postoperative drainage amount within 16 hours was higher in the placebo group (409 ± 134 mL) than in the topical group (222 ± 85 mL, *p* < 0.001) and the IV group (304 ± 147 mL, *p* = 0.006). The total drainage amount was also higher in the placebo group than in the topical and IV groups (626 ± 149 mL versus 412 ± 122 mL versus 525 ± 185 mL, *p* < 0.001 and *p* = 0.041, resp.).

The total blood loss was 1131 mL (range, 567–1845 mL) in the placebo group, which was higher than that in the topical group (795 mL; range, 336–1350 mL; *p* < 0.001) and the IV group (921 mL; range, 465–1495 mL; *p* = 0.014). The blood conservation effect was 29.7% if TXA was administered via the topical route and 18.5% if TXA was administered via the IV route. However, there was no difference in total blood loss between the topical group and the IV group (*p* = 0.179). With regard to allogeneic blood transfusion, 2 of the 30 patients in the placebo group (6.7%) required a red blood cell transfusion, each needing one unit on POD2. No transfusions (0%) were required in the topical and IV groups. There were no statistical differences in the transfusion rate, VAS on POD1, length of hospital stay, and the range of motion of the knee on POD 14 between the 3 groups (Table 2).

The occurrence of wound complications, including ecchymosis of the affected limb, hematoma, or infection of the knee, did not differ between the 3 groups (Table 3). An 81-year-old man in the IV group developed a symptomatic DVT, and an ascending venogram of the leg showed thrombosis in the peroneal vein. The leg edema resolved after warfarin therapy at the cardiology clinic for 3 months. There were no differences in the incidence of symptomatic DVT between the 3 groups, nor was symptomatic pulmonary embolism observed in any of the patients during clinical follow-up for 3 months (Table 3).

One patient in the topical group sustained a deep periprosthetic infection 2 weeks after surgery, which required further surgery for debridement and systemic antibiotic therapy, resulting in a satisfactory outcome. However, in terms of return to operating theater because of wound complications, there were no differences between the 3 groups (Table 3). One 83-year-old woman in the topical group died of endemic Dengue fever after she was discharged from the hospital. There were no deaths in the 3 groups owing to venous thromboembolism (VTE) or postoperative bleeding in the current study.

## 5. Discussion

Our prospective, randomized, double-blind trial demonstrated that both topical and systemic administration of TXA significantly reduced postoperative bleeding after minimally invasive TKA in patients receiving rivaroxaban for thromboprophylaxis. The mean reduction in total blood loss was 29.7% in the topical TXA group and 18.5% in the IV TXA group; these results were comparable with those of our previous study, which showed a blood conservation effect of 15% and 19%, respectively, in minimally invasive TKA patients receiving low-molecular-weight-heparin (LWMH) for thromboprophylaxis [11]. The total blood loss calculated in our previous study was 1222 mL versus 1035 mL versus 986 mL in the placebo group, one-TXA (1 g intraoperative) group and two-TXA (1 g preoperative and 1 g intraoperative) group, respectively [11]. In the current study, the total blood loss was calculated to be 1131 mL versus 921 mL versus 795 mL in the placebo group, intravenous group, and topical TXA group, respectively. The blood conservation effect of TXA was similar in minimally invasive TKA patients when administered by the topical or systemic route. One other finding was that rivaroxaban, a direct oral anticoagulant, did not increase the postoperative bleeding, as demonstrated by comparison with the results of our previous study using LMWH (1131 mL versus 1222 mL) in minimally invasive TKA patients [11].

Regarding the formula of intraoperative injection solution, there is no consensus. In our study, we used the formula of 3 g TXA in 100 ml saline as Soni et al. [32] and Maniar et al. [33] and our final volume of this formula was 160 ml. The VAS on POD1, and the range of motion of the knee were not different between the three groups and not different from our previous study [10, 11]. No adverse effects such as increase postoperative pain and delay of postoperative rehabilitation were noted after intraarticular injection.

The finding of an equal efficacy of topical and intravenous TXA administration in terms of reducing postoperative blood loss during TKA was consistent with the results of other studies that employed topical TXA administration at dose of 1.5 g, 2 g, or 3 g [10, 34–36]. However, with regard to the transfusion rate, discrepancies exist between reports, which may be related to the different doses of TXA administered topically, different criteria used to indicate the necessity of blood transfusion, or the surgical technique employed (i.e., standard or minimally invasive TKA). Konig et al. [37] reported a reduction in blood loss (1729 mL versus 1384 mL) and a lower transfusion rate (10% versus 0%) after topical administration of 3 g TXA in standard TKA patients as compared with the control group. The mean blood loss of 1131 ± 336 mL calculated in this study for the placebo group was lower than that reported in other studies of standard TKA patients, which may be related to employment of the minimally invasive technique. The transfusion rates in the placebo, IV TXA, and topical TXA groups in this study were 6.7%, 0%, and 0% (*p* = 0.102), respectively, which were extremely low as compared with a study by Seo et al. [38] who reported transfusion rates of 94%, 34%, and 20% in placebo, IV TXA, and topical TXA groups, respectively. This high discrepancy may result from the use of different criteria indicating the necessity of a blood transfusion, in addition to the different surgical technique.

Previous studies have demonstrated that anticoagulants, which reduce the incidence of DVT, are associated with an increased risk of postoperative bleeding [12, 32, 39]. Modern direct oral anticoagulants, such as rivaroxaban and dabigatran, have been shown to be associated with a higher risk of postoperative bleeding than enoxaparin, a kind of LMWH, after joint replacement surgery based on a meta-analysis of 4 clinical trials [40]. Increased postoperative bleeding after rivaroxaban therapy for thromboprophylaxis in TKA and THA patients was reportedly related to increased wound complications, a higher infection rate (from 1% to 2.5%), and a longer hospital stay [41]. Our data did not show any differences in the incidences of wound complications or infection between the placebo group and the TXA groups, patients in all groups being given rivaroxaban. We considered that this may relate to the lower total blood loss in the placebo group (1132 mL) achieved by use of a minimally invasive technique as compared with previous studies in which the standard technique was employed [37, 41, 42] and a small patient group in our study.

Data from clinical trials and meta-analyses have shown that systemic administration of TXA does not lead to an increase in the incidence of DVT or VTE [12, 13, 15]. Wong et al. demonstrated that the plasma level of TXA after topical application was significantly (~70%) lower than that following an equivalent dose of TXA via systemic administration [17]. Therefore, topical intra-articular application of TXA during TKA is safer in terms of thromboembolic risk than systemic administration. In a previous study, Doppler ultrasonography was performed systemically in patients 2 to 3 days after TKA, and the results showed no increase in the rate of thromboembolism after topical TXA application [17]. In the current study, no difference in the occurrence of DVT was noted in patients with or without TXA administration. No other VTEs were observed neither.

In conclusion, the results of our prospective, randomized, double-blind trial showed that topical (intra-articular) and intravenous administration of TXA are of equal efficacy in terms of reducing postoperative blood loss after minimally invasive TKA in patients receiving rivaroxaban for thromboprophylaxis. No increased thromboembolic risks were noted.

## Figures and Tables

**Table 1:** Characteristics of the patients.

Characteristics	Placebo (*n* = 30)	IV (*n* = 31)	Topical (*n* = 32)	*p*
Age (yr) (SD; range)	70.87 (6.05; 59–81)	69.13 (7.94; 51–85)	69.66 (5.53; 59–84)	0.575
BMI (kg/m^2^) (SD; range)	28.26 (4.84; 19.9–39.8)	28.14 (4.53; 20.6–42.1)	28.40 (5.38; 21.2–49.0)	0.980
Women (%)	24 (80.0%)	27 (87.1%)	19 (59.4%)	0.029
Preoperative Hb (g/dL) (SD; range)	13.04 (1.03; 9.5–15.3)	13.37 (0.98; 9.5–16.8)	13.27 (1.35; 11.1–16.8)	0.518
Preoperative Hct (%) (SD; range)	39.44 (2.78; 30.0–45.3)	40.08 (2.97; 30.0–50.8)	39.60 (3.67; 32.4–48.1)	0.551
PT (SD; range)	10.31 (0.36; 9.6–11.1)	10.21 (0.41; 9.5–12.2)	10.31 (0.44; 9.7–12.2)	0.611
APTT (SD; range)	27.27 (2.12; 22.9–32.8)	26.97 (2.32; 21.3–33.1)	26.89 (2.02; 22.8–33.1)	0.763
Platelet count (1000/*μ*L) (SD; range)	225.30 (50.85; 125–308)	231.26 (72.09; 106–442)	229.50 (46.70; 151–337)	0.918
ASA—number/total number (%)				0.210
I	1/30 (3.3%)	1/31 (3.2%)	0/32 (0.0%)	
II	24/30 (80.0%)	22/31 (71.0%)	19/32 (59.4%)	
III	4/30 (13.3%)	8/31 (25.8%)	13/32 (40.6%)	

SD: standard deviation; APTT: activated partial thromboplastin time; ASA: American Society of Anesthesiologists; BMI: body mass index; INR: interactional normalized ratio; PT: prothrombin time; Hb: hemoglobin; Hct: hematocrit; *p* > 0.005 for all characteristics of patients between all groups, with the exception of gender distribution, the percentage of female patients in the topical group being lower than the percentages in the placebo and IV groups (*p* = 0.029).

**Table 2:** Postoperative data for all patients.

Event	Placebo (*n* = 30)	IV (*n* = 31)	Topical (*n* = 32)	*p*		
				Placebo versus Topical	Placebo versus IV	Topical versus IV
Wound length on extension (cm)	8.66 (0.923; 7–11)	8.66 (1.143; 7–12)	8.61 (0.998; 7–11)	0.265	0.265	0.261
Postoperative Hb level						
POD1 (g/dl)	11.02 (1.06; 8.6–13.2)	11.57 (1.26; 9.7–15.5)	12.04 (1.28; 9.8–14.7)	0.005	0.214	0.304
POD2 (g/dl)	9.73 (0.98; 7.2–11.4)	10.46 (1.27; 8.8–15.1)	11.28 (1.26; 8.8–14.1)	<0.001	0.063	0.025
POD4 (g/dl)	9.23 (1.11; 7.4-11.1)	10.06 (1.29; 8.0–13.8)	10.64 (1.34; 8.4–14.3)	<0.001	0.043	0.193
Postoperative drainage (mL)						
0–16 hours	409 (134; 180–810)	304 (147; 75–700)	222 (85; 20–345)	<0.001	0.006	0.036
0–48 hours	626 (149; 260–1020)	525 (185; 220–1000)	412 (122; 145–610)	<0.001	0.041	0.018
Blood transfusion rate (number/total number)	6.7% (2/30)	0.0% (0/31)	0.0% (0/32)	0.102		
Total blood loss (mL)	1131 (336; 567–1845)	921 (252; 465–1495)	795 (231; 336–1350)	<0.001	0.014	0.197
*VAS on POD1*	*3.89 (0.83; 3*–*6)*	*3.84 (0.74; 3*–*6)*	*3.93 (0.84; 3*–*6)*	*0.949*		
Length of stay (days)	6 (5–8)	6 (5–8)	5.9 (5–7)	0.280		
*Range of motion on POD 14*	*109.1 (11.8; 90–145)*	*105.7 (12.9; 60–135)*	*109.2 (6.7; 95–120)*	*0.419*		

Continuous data are presented as mean (standard deviation, range); POD: postoperative day; *VAS: visual analogue scale.*

**Table 3:** Safety outcomes.

Event	Placebo (*n *= 30)	IV (*n* = 31)	Topical (*n *= 32)	*p*
Up to 3 months				
Pulmonary embolism	0/30	0/31	0/31	
Symptomatic deep-vein thrombosis	0/30	1/31	0/31	
Return to OR because of wound complication (deep infection)	0/30 (0.0%)	0/31 (0.0%)	1/31 (3.1%)	1.000
Ecchymosis	7/30 (23.3%)	4/31 (12.9%)	4/31 (12.5%)	0.244
*Hematoma*	3/30 (10%)	6/31 (19.4%)	5/31 (15.6%)	0.308
Death	0/30 (0.0%)	0/31 (0.0%)	1/32 (3.1%)^*∗*^	1.000

^*∗*^One patient in the topical group died of endemic Dengue fever after discharge from the hospital; ^*∗*^OR: operating theater.
